# CYLD stimulates macrophage phagocytosis of leukemic cells through STAT1 signalling in acute myeloid leukemia

**DOI:** 10.1371/journal.pone.0283586

**Published:** 2023-08-07

**Authors:** Nguyen Thanh Huyen, Nguyen Thy Ngoc, Nguyen Hoang Giang, Do Thi Trang, Ha Hong Hanh, Vu Duc Binh, Nguyen Van Giang, Nguyen Xuan Canh, Nguyen Thi Xuan

**Affiliations:** 1 Graduate University of Science and Technology, Vietnam Academy of Science and Technology, Cau Giay, Ha Noi, Vietnam; 2 Faculty of Biotechnology, Vietnam National University of Agriculture, Gia Lam, Hanoi, Vietnam; 3 University of Science and Technology of Hanoi, Vietnam Academy of Science and Technology, Cau Giay, Ha Noi, Vietnam; 4 Institute of Genome Research, Vietnam Academy of Science and Technology, Cau Giay, Hanoi, Vietnam; 5 National Institute of Hematology and Blood Transfusion, Pham Van Bach, Ha Noi, Vietnam; Aristotle University of Thessaloniki School of Biology, GREECE

## Abstract

Acute myeloid leukemia (AML) is the most aggressive hematopoietic malignancy characterized by uncontrolled proliferation of myeloid progenitor cells within the bone marrow. Tumor suppressor cylindromatosis (CYLD) is a deubiquitinating enzyme, which suppresses inflammatory response in macrophages. Macrophages have a central role in the defense against foreign substances and circulating cancer cells by their professional phagocytic capacity. Little is known about contributions of CYLD to changes in biological properties of human macrophages and its involvement in AML. The present study, therefore, explored whether macrophage functions in healthy individuals and AML patients are influenced by CYLD. To this end, ninety-two newly diagnosed AML patients and 80 healthy controls were recruited. The mRNA expression levels of inflammation-related genes were evaluated by real-time PCR, cell maturation, phagocytosis and apoptosis assays by flow cytometry and secretion of inflammatory cytokines by ELISA. As a result, AML patients with the low CYLD expression were significantly higher in M4/M5 than other subtypes according to the FAB type. The low CYLD expression was also closely associated with older patients and enhanced level of LDH in AML. Moreover, treatment of normal macrophages with CYLD siRNA enhanced activation of STAT-1, leading to increases in expressions of maturation markers and IL-6 production as well as suppression in cell apoptosis and phagocytosis, while macrophage phagocytosis from AML M4/M5b was higher than that from healthy controls upon CYLD siRNA transfection through STAT1 signalling. In conclusion, the inhibitory effects of CYLD on macrophage functions are expected to affect the immune response in AML.

## Introduction

Acute myeloid leukemia (AML) is the most aggressive hematopoietic malignancy characterized by uncontrolled proliferation and prolonged survival of myeloid progenitor cells within the bone marrow [1]. The most commonly mutated gene in AML is FMS-like tyrosine kinase 3 receptor (FLT3), which is overexpressed in leukemic cells [2]. Activation of FLT3 leads to cancer cell survival and proliferation through inflammation-mediated pathways including the Janus kinase/signal transducer and activator of transcription (JAK/STAT) and nuclear factor kappa B (NF-κB) [3], in which constitutive activations of STAT1 [4], STAT3 and STAT5 [1] are detected in patients with myeloid leukemia. High levels of TNF-α and IL-6 cytokines are correlated with more severe fatigue and aggressive disease [5], whereas anti-inflammatory cytokines such as TGF-β and IL-10 contribute to their suppressive activity to inhibit AML progression [6]. In AML, monocytic and myelomonocytic leukemias exhibit marked phagocytosis which distinguished them from the other AML subtypes [7]. Patients with AML have a high level of serum lactate dehydrogenase (LDH), which is used for diagnosis and prognosis of AML [8]. LDH is also a marker of immune suppression and associated with the risk of developing severe hematologic neoplasms [9].

Tumor suppressor cylindromatosis (CYLD) is a deubiquitinating enzyme, which negatively regulates activation of NF-κB and STAT pathways to suppress inflammatory diseases [10,11]. CYLD is also known a tumor suppressor that is identified in familial cylindromatosis and Brooke-Spiegler syndrome, due to inherited CYLD gene mutation [12]. Attenuated expression of CYLD is correlated with a poor prognosis in multiple myelomas [13] and leukemia/lymphoma [11,14]. In mice, CYLD in macrophages inhibits antibacterial immune responses [10].

Macrophages are professional phagocytic cells in the innate immune response and have a central role in the defense against foreign substances and circulating cancer cells [15]. Macrophage phagocytosis is not only to engulf pathogens but also to present their antigens for stimulating the anti-tumor immune responses [16]. Macrophage differentiation is induced by macrophage colony-stimulating factor (M-CSF), which is highly expressed in AML cells [17]. Macrophages are remarkably increased in bone marrow biopsies samples of AML patients [18]. When activated, macrophages lose their phagocytic efficiency [19]. In acute monocytic leukemia, the phagocytic ability of macrophages is sensitive to CD47 [20].

Little is known about the regulatory roles of CYLD on functions of human macrophages and involvement of STAT signallings in regulating macrophage activation in AML. To this end, macrophages were exposed to LPS in the presence or absence of CYLD siRNA and expression of maturation markers, cytokine release, and phagocytic capacity were examined. Activities of STATs were assessed in LPS-stimulated macrophages to determine the mechanism underlying the regulation of CYLD in macrophages. The eliminating effects of leukemic cells by macrophages in AML patients were also determined.

## Materials and methods

### Patients and control subjects

Ninety-two newly diagnosed AML patients from August 2020 to February 2023 and 80 healthy controls were recruited into the study at the National Institute of Hematology and Blood Transfusion, Ha Noi, Vietnam. The diagnosis of AML was based on the 2016 WHO criteria [21] and the traditional French-American-British (FAB) classification [22], in which the cytomorphological features of peripheral blood and bone marrow cells are used to classify the different subtypes of AML. According to FAB type, 92 AML patients were diagnosed as M1 (n = 10), M2 (n = 20), M3 (n = 10), M4 (n = 32) and M5 (n = 20), respectively. No individuals in the control population took any medication or suffered from any known acute or chronic disease. All volunteers gave a written consent to participate in the study. Person care and experimental procedures were performed according to the Vietnamese law for the welfare of human and were approved by the Ethical Committee of Institute of Genome Research, Vietnam Academy of Science and Technology.

### Cell culture

Peripheral blood mononuclear cells (PBMCs) from whole blood samples of healthy donors and bone marrow (BM) cells of AML patients were collected and transferred to sterile tubes containing EDTA as anticoagulant. The cells were isolated via density gradient centrifugation (Ficoll-Paque Plus, GE Healthcare Life Sciences). Freshly isolated PBMCs and BM cells were obtained by centrifuging at 400g for 30 min at room temperature. The cells were cultured for 6 days in RPMI-1640 (GIBCO) containing: 10% FCS, 1% penicillin/streptomycin, 1% glutamine and 1% non-essential amino acids (NEAA) at a density of 5×10^6^ cells/ml. Cultures were supplemented with M-CSF (50ng/mL, **Peprotech)** on days 0 and 4 to attain PBMC- (MDMs) or BM-derived macrophages (BMDMs). For maturation, macrophages were cultured with lipopolysaccharide (LPS, 1°g/ml, Sigma Aldrich) for 24 hours.

### Transfection of cells with siRNA

Human CYLD- and STAT1- targeted and control siRNAs (pre-designed siRNA, Thermo Fisher Scientific) were transfected into cells (5 x 10^**6**^ cells/ml) with the help of Lipofectamine 3000 (Thermo Fisher Scientific) according to the manufacturer’s recommendations. Cells were incubated for 48h at 37^**°**^C, 5% CO_**2**_ and followed by treatment with LPS (1°g/ml, Sigma Aldrich) or with fludarabine (80 °M, Sigma Aldrich). After washing three times with PBS, the cells were used for further experiments.

### RNA extraction and real-time RT-PCR

Total mRNA was isolated using the Qiashredder and RNeasy Mini Kit from Qiagen according to the manufacturer’s instructions. For cDNA first-strand synthesis, 1 °g of total RNA in 12.5 °l DEPC-H_**2**_O was mixed with 1 °l of oligo-dT primer (500 °g/ml, Invitrogen) and heated for 2 min at 70°C. To determine transcript levels of CYLD, STAT-1, STAT-3, STAT-5, STAT-6, SHP-1, SHP-2 and GAPDH, the quantitative real-time PCR with the LightCycler System (Roche Diagnostics) was applied. The following primers were used: CYLD primers: 5’-TGCCTTCCAACTCTCGTCTTG-3’ (forward) and 5’-AATCCGCTCTTCCCAGTAGG-3’ (reverse); STAT-1 primers: 5’-CCCTTCTGGCTTTGGATTGAA-3’ (forward) and 5’-CTTCCCGGGAGCTCTCACTGA-3’ (reverse); STAT-3 primers: 5’-GGA GGA GTT GCA GCA AAA AG-3’ (forward) and 5’-TGT GTT TGT GCC CAG AAT GT-3’ (reverse); STAT-5 primers: 5’-CAGACCAAGTTTGCAGCCAC-3’ (forward) and 5’-CACAGCACTTTGTCAGGCAC-3’ (reverse); STAT-6 primers: 5’-GCCCACTCACTCCAGAGGACCT-3’ (forward) and 5’-GGTGTTGGGGAAAGTCGACAT-3’ (reverse); SHP1 primers: 5’- GCCCAGTTCATTGAAACCAC-3’ (forward) and 5’- GAGGGAACCCTTGCTCTTCT-3’ (reverse); SHP2 primers: 5’GAGAGCAATGACGGCAAGTCT3’ (forward) and 5’- CCTCCACCAACGTCGTATTTC-3’ (reverse); and GAPDH primers: 5’-GGAGCGAGATCCCTCCAAA-3’ (forward) and 5’-GGCTGTTGTCATACTTCTCAT-3’ (reverse). PCR reactions were performed in a final volume of 20 °l containing 2 °l cDNA, 2.4 °l MgCl_**2**_ (3 °M), 1 °l primer mix (0.5 °M of both primers), 2 °l cDNA Master SybrGreen I mix (Roche Molecular Biochemicals), and 12.6 °l DEPC-treated water. The target DNA was amplified during 40 cycles of 95°C for 10 s, 62°C for 10 s, and 72°C for 16 s, each with a temperature transition rate of 20°C/s, a secondary target temperature of 50°C, and a step size of 0.5°C. Melting curve analysis was performed at 95°C, 0 s; 60°C, 10 s; 95°C, 0 s to determine the melting temperature of primer dimers and the specific PCR products. The ratio between the respective gene and corresponding GAPDH was calculated per sample according to the ΔΔ cycle threshold method [23].

### Immunostaining and flow cytometry

Cells (5×10^**5**^) were incubated in 100 °l FACS buffer (phosphate buffered saline (PBS) plus 0.1% FCS) containing fluorochrome-conjugated antibodies at a concentration of 10 °g/ml. The following antibodies were used for staining: human IgG isotype control, anti-human CD11b, CD40, CD68, and CD86 (all from eBioscience). After incubating with the antibodies for 60 min at 4^**°**^C, the cells were washed twice and resuspended in FACS buffer for flow cytometry analysis (FACSAria Fusion, BD Biosciences).

### Western blotting

MDMs (3 x 10^**6**^ cells each) were washed twice in PBS, and lysed in RIPA-1 buffer. Lysates were stored at -80°C until used for western blotting. The lysates were separated by 10% SDS-polyacrylamide gels, and blotted on polyvinylidene fluoride membrane. The blots were blocked with blocking buffer. Then the blots were probed overnight with anti-p-STAT-1 (727) and anti-GAPDH (Santa Cruz) in blocking buffer, washed 5 times, probed with HRP anti-rabbit or anti-mouse secondary antibody (Amersham) for 1h at RT, and washed final 5 times. Antibody binding was detected with the enhanced ECL Plus kit (GE Healthcare).

### Cytokine quantification in cell supernatants

Serum was isolated from the blood samples and cell supernatants were collected from the cell culture. Serum and cell supernatant were stored at -20°C until use for ELISA. TNF-α, IL-6, IL-1β and IFN-γ were determined by using ELISA kits (Thermo Scientific) according to the manufacturer’s protocol.

### Phagocytosis assay

FITC-dextran uptake assay: Cells (2 x 10^5^ cells/ml) were suspended in prewarmed serum-free RPMI 1640 medium, pulsed with FITC-conjugated dextran (Sigma-Aldrich) at a final concentration of 1 mg/ml and incubated for 3h at 37^°^C. Uptake was stopped by adding ice-cold PBS. Then the cells were washed three times with FACS buffer containing 0.01% sodium azide. The cells were analysed for the uptake of FITC-dextran with flow cytometry (FACSAria Fusion, BD Biosciences).

Phagocytosis of leukemic cell assay: Cells were cultured for 3h with carboxyfluorescein diacetate succinimidyl ester (CFSE)-labeled AML cells at the ratio of 1:2 and then washed three times with PBS. Subsequently, the cells were stained with anti-CD68 for 60 min at 4°C, washed twice and resuspended in FACS buffer for flow cytometry analysis. Phagocytosing cells were defined as the percentage of CD68^+^CFSE^+^ cells.

### Phosphatidylserine translocation and propidium iodide incorporation

The presence of phosphatidylserine (PS) on the outer surface of apoptotic cells was detected with fluorescein isothiocyanate (FITC)-conjugated annexin V binding to PS at the cell surface and necrotic cells were assessed from the amount of propidium iodide (PI)-positive cells. In brief, 10^5^ cells were harvested and washed twice with Annexin washing buffer (AWB). The cell pellet was resuspended in 100 °l of Annexin V/PI labelling solution (eBioscience), and incubated for 15 min at room temperature. After washing with AWB, the cells were analysed by flow cytometry.

### Statistics

Statistical analysis was performed with the SPSS and GraphPad Prism 8 softwares. The statistical significance of the differences was determined by the two-sided unpaired Student’s t test for the comparisons between two groups; for more than two groups, the one-way ANOVA analysis of variance was used. P<0.05 was considered statistically significant.

## Results

### Correlation between CYLD expression in AML subtypes and clinical outcomes

**Fig 1:**
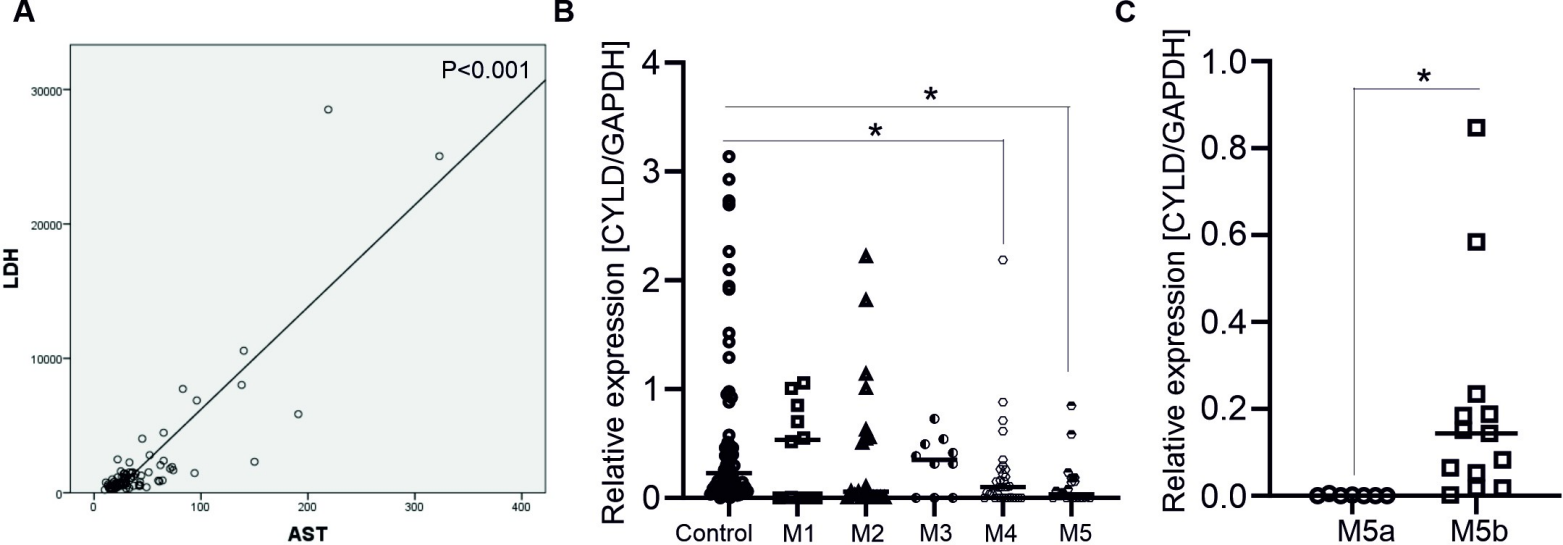
Correlation between CYLD expression and clinical outcomes in AML. A. The Spearman correlation analysis shows the expression levels of LDH and AST (n  =  92) in AML patients. *** p<0.001 (Unpaired two-tailed t-test). B. Graph indicates the mRNA level of CYLD in AML M1 (n = 10), M2 (n = 20), M3 (n = 10), M4 (n = 32) and M5 (n = 20) subtypes; each dot represents a single sample. * (p<0.05) shows significant difference from healthy individuals (ANOVA). C. Graph indicates the mRNA level of CYLD in AML M5a (n = 7) and M5b subtypes (n = 13); each dot represents a single sample. * (p<0.05) shows significant difference from AML M5a subtype (Unpaired two-tailed Student’s t-test).

As shown in Table 1, the mean age of the AML patients was 46.2 years and the gender distribution was 50 males and 42 females. Clinical profile showed significant increases in glucose, ferritin, AST, GGT and LDH concentrations in the patient group. Besides, a positive correlation between AST and LDH levels was observed in these patients (P<0.001) (Fig 1A).

**Table 1:** Association between CYLD expression and clinical parameters in AML patients.

Characteristic	Normal range	Total	CYLD		*p* value
Number of patients		n = 92	Low (n = 67)	High (n = 25)	
Age (years)		46.2 (1–84)	59.1 (20–84)	30.1 (1–60)	**<0.001**
Sex, male (n, %)		50 (54.3)	32 (47.77)	16 (76)	
Urea (mmol/l)	7.5	6.14±0.35	6.04 ± 0.47	6.2 ± 0.53	0.84
Glucose (mmol/l)	6.4	**6.46 ± 0.22**	6.41 ± 0.28	6.67 ± 0.34	0.61
Creatinine (°mol/l)	120 (M)/100 (F)	90.53 ±4.28	90.8 ± 6.3	92.27 ± 5.6	0.888
Uric acid (°mol/l)	420 (M)/360 (F)	411.18 ± 16.9	425.36± 22.85	392.88± 29.3	0.423
Total bilirubin (°mol/l)	17	14.26 ± 1.58	13.85 ± 1.69	11.93 ± 1.04	0.484
Direct bilirubin (°mol/l)	4.3	4.24 ± 1.01	4.06 ± 1.12	2.67 ± 0.5	0.43
Indirect bilirubin (°mol/l)	12.7	10.11 ± 0.64	9.94± 0.7	9.31 ± 0.67	0.59
Total protein (g/l)	82	78.34 ± 4.35	80.46 ± 6.55	73.77 ± 1.31	0.521
Albumin (g/l)	50	37.95 ± 0.53	38.12 ± 0.71	37.98 ± 0.72	0.9
Globulin (g/l)	38	36.06 ± 0.81	35.78 ± 0.94	35.79 ± 1.27	0.99
Ferritin (°g/l)	400	**945.13 ± 67.8**	1013.09 ± 84.83	934.8 ± 132.5	0.62
AST (GOT) (U/l)	37	**44.2 ± 4.63**	49.56 ± 6.73	33.15 ± 3.85	0.134
ALT (GPT) (U/l)	40	37.46 ± 3.97	39.68 ± 5.54	35.44 ± 5.65	0.65
GGT (UI/L)	60	**90.28 ± 12.14**	85.27 ± 13.22	103± 23.11	0.5
LDH (U/l)	460	**1879.17 ± 413**	2045.1 ± 428.5	671.88 ± 64	**0.043**
Relative expression of STAT1	1.02±0.11	1.097±0.47	1.303±0.67	0.577±0.11	0.319
Relative expression of STAT3	0.9±0.17	1.42±0.64	1.53±0.92	1.33±0.297	0.893

Since aberrant expression of CYLD is found frequently in leukemia [14], we ask whether there is a correlation between CYLD expression level and clinical outcomes. The expression of CYLD in AML patients was divided into two groups based on the median CYLD expression value in healthy controls (high vs. low). The high CYLD expression group was detected in 25 samples (27.17%) and the low CYLD expression group was detected in 67 samples (72.83%, Table 1). The relationship between CYLD expression and clinical features at diagnosis was analysed. Results indicated that patients with the low CYLD expression, which were more likely in elderly than young patients and had a significant elevation of LDH compared to those with the high CYLD expression (Table 1). AST level was slightly higher in the low CYLD expression group (49.56 ± 6.73 U/I) than that in the high CYLD expression group (33.15± 3.85 U/I) (p = 0.134) (Table 1). Moreover, STAT1 expression, but not STAT3 tended to be higher than in the low CYLD expression group (Table 1). The evidences suggested that the low CYLD expressing group frequently occurred in the older patients and affected the release of serum LDH and partially AST in AML. In addition, the significant differences in other clinical indicators between the two groups did not exist (Table 1).

Importantly, patients with AML M4 and M5, but not the other subtypes had significantly lower level of CYLD than normal controls (Fig 1B). More importantly, the frequency of M5a group with the low CYLD expression was demonstrated in 100% and its level was even significantly lower in the M5a compared to M5b groups (Fig 1C). Discrepancies in CYLD expression between the five subgroups reflected that the low CYLD expression was at a higher risk for AML M4 or M5 progression.

### CYLD inhibits STAT1 signalling in MDMs

**Fig 2:**
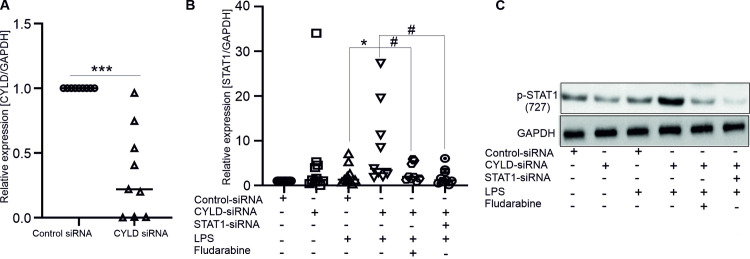
Effects of CYLD on STAT1 signallings in human macrophages (MDMs). A. Graph indicates the mRNA level of CYLD in control siRNA- and CYLD siRNA -treated MDMs (n = 10). *** (p<0.001) indicates significant difference from control siRNA-treated MDMs (Unpaired two-tailed t-test). B. Graph indicates the mRNA level of STAT1 in control siRNA- and CYLD siRNA -treated MDMs (n = 7–10), which were unstimulated or stimulated with LPS in the presence or absence of fludarabine or STAT1 siRNA. *(p<0.05) indicates significant difference from control siRNA-treated MDMs; # (p<0.05) indicates significant difference from CYLD-silenced LPS-treated MDMs (ANOVA). C. Representative western blot images (n = 5) of control siRNA- and CYLD siRNA-treated MDMs, which were unstimulated or stimulated with LPS in the presence or absence of fludarabine or STAT1 siRNA. Protein extracts were analysed by direct Western blotting using antibodies directed against p-STAT-1 (727) and GAPDH.

As LPS is recognized by toll-like receptor 4 (TLR4) to trigger transcription of multiple genes associated with the regulation of maturation/differentiation and activation of macrophages [24], we examined whether CYLD contributes to the regulatory effects on LPS‐induced functions of human macrophages. The cells were transfected with control or CYLD siRNA in the presence or absence of LPS. It is concluded that the mRNA level of CYLD was down-regulated in CYLD-silenced MDMs (Fig 2A). As shown in Fig 2B and 2C, treatment of MDMs with control or CYLD siRNA significantly increased the mRNA expression and phosphorylation of STAT1 upon LPS treatment and did not affect the expression levels of STAT3, STAT5, STAT6, SHP1 and SHP2 (S1 Fig). Therefore, CYLD significantly contributed as an inhibitor of activation of STAT1 signalling in MDMs.

### CYLD inhibits MDM functions through STAT-1 signalling

**Fig 3:**
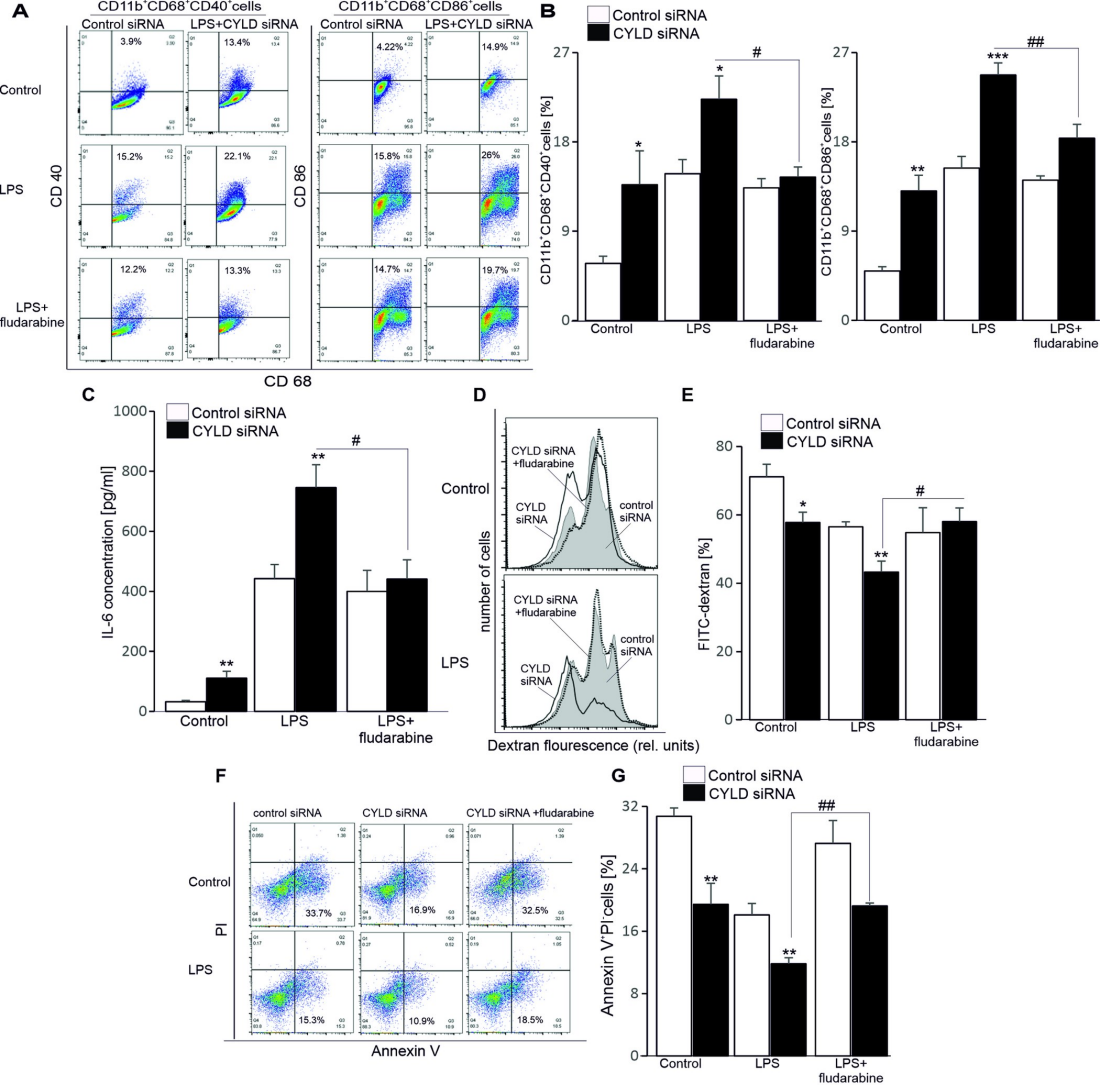
Effects of CYLD on MDM functions through STAT-1 signalling. A. Representative dot plots (n = 7) of CD11b+CD68+CD40+ and CD11b+CD68+CD86+ (gated with CD11b+) cells, which are transfected with control siRNA and CYLD siRNA and followed by LPS treatment in the presence or absence of fludarabine. B. Arithmetic means ± SEM (n = 7) of percentages of CD11b+ CD68+CD40+ and CD11b+ CD68+CD86+ cells, which are transfected with control siRNA (white bars) and CYLD siRNA (black bars) and followed by LPS treatment in the presence or absence of fludarabine. *(p<0.05), ** (p<0.01) and *** (p<0.001) indicate significant differences from control siRNA-treated MDMs; #(p<0.05) and ##(p<0.05) indicate significant differences from LPS-treated MDMs (ANOVA). C, E and G. Arithmetic means ± SEM (n = 5–8) of IL6 level by MDMs (C) or percentages of MDMs stained by FITC-dextran (E) or expressed Annexin V+PI- (G), which are transfected with control siRNA (white bars) and CYLD siRNA (black bars) and followed by LPS treatment in the presence or absence of fludarabine. *(p<0.05) and **(p<0.01) indicate significant differences from control siRNA-treated MDMs; #(p<0.05) and ##(p<0.05) indicate significant differences from LPS-treated MDMs (ANOVA). D. Representative FACS histograms (n = 6) depicting FITC-dextran accumulation by MDMs, which are transfected with control siRNA and CYLD siRNA and followed by LPS treatment in the presence or absence of fludarabine. F. Representative dot plots (n = 8) of Annexin V^**+**^PI^**-**^ cells, which are transfected with control siRNA and CYLD siRNA and followed by LPS treatment in the presence or absence of fludarabine.

To ask whether MDM functions is regulated by CYLD through STAT-1 signalling, cells were transfected with control or CYLD siRNA for 48h and followed by treatment with a pharmacological inhibitor of STAT-1 signalling fludarabine in the presence or absence of LPS for another 24 h. Cells were collected and stained for CD11b, CD68, CD40 and CD86. The CD11b^**+**^CD68^**+**^gated population was analysed for the expression of costimulatory molecules CD40 and CD86. As shown in Fig 3A and 3B, LPS treatment increased the percentages of CD86^**+**^ and CD40^**+**^ expressing MDMs, which were further elevated in the absence of CYLD. Importantly, upregulation of CD86 and CD40 by CYLD-silenced mature MDMs was blunted in the presence of fludarabine.

We next examined cytokine productions secreted by MDMs. LPS treatment leads to increased release of TNF-α, IL-6, IL-1β and IFN-γ cytokines (Figs 3C and S2), however, CYLD-silenced MDMs produced higher IL-6 only as compared to control siRNA-treated MDMs and IL-6 level was unaffected when fludarabine was present in the cell culture (Fig 3C).

In addition to maturation and the secretion of cytokine productions, macrophages are capable to phagocytose foreign particles and cancer cells, which are assessed by FITC-dextran uptake and phagocytosis of CFSE-treated AML cells. Macrophage maturation reduces their efficacy of phagocytosis [19]. As expected, treatment of MDMs with CYLD siRNA significantly decreased FITC-dextran uptake (Fig 3D–3E) and slightly prevented the phagocytosis of CFSE-treated AML cells (S3 Fig). In the presence of fludarabine, the promoting effect of CYLD on FITC-dextran uptake was abolished in MDMs (Fig 3D–3E).

Phagocytosis of pathogens lead to apoptosis of macrophages [25]. Hence, we investigated whether altered CYLD expression affects the survival of normal macrophages. Accordingly, the percentage of apoptotic (Annexin V^**+**^PI^**-**^) cells were determined by flow cytometry. As expected, treatment of MDMs with CYLD siRNA significantly decreased the number of Annexin V^**+**^PI^**-**^ (Fig 3F–3G), the effect was also abrogated in the presence of fludarabine (Fig 3F–3G), demonstrating that the promoting effects of CYLD on FITC-dextran uptake and apoptosis of MDMs required STAT-1 signalling.

### Effects of CYLD on macrophage phagocytosis in AML subtypes

**Fig 4:**
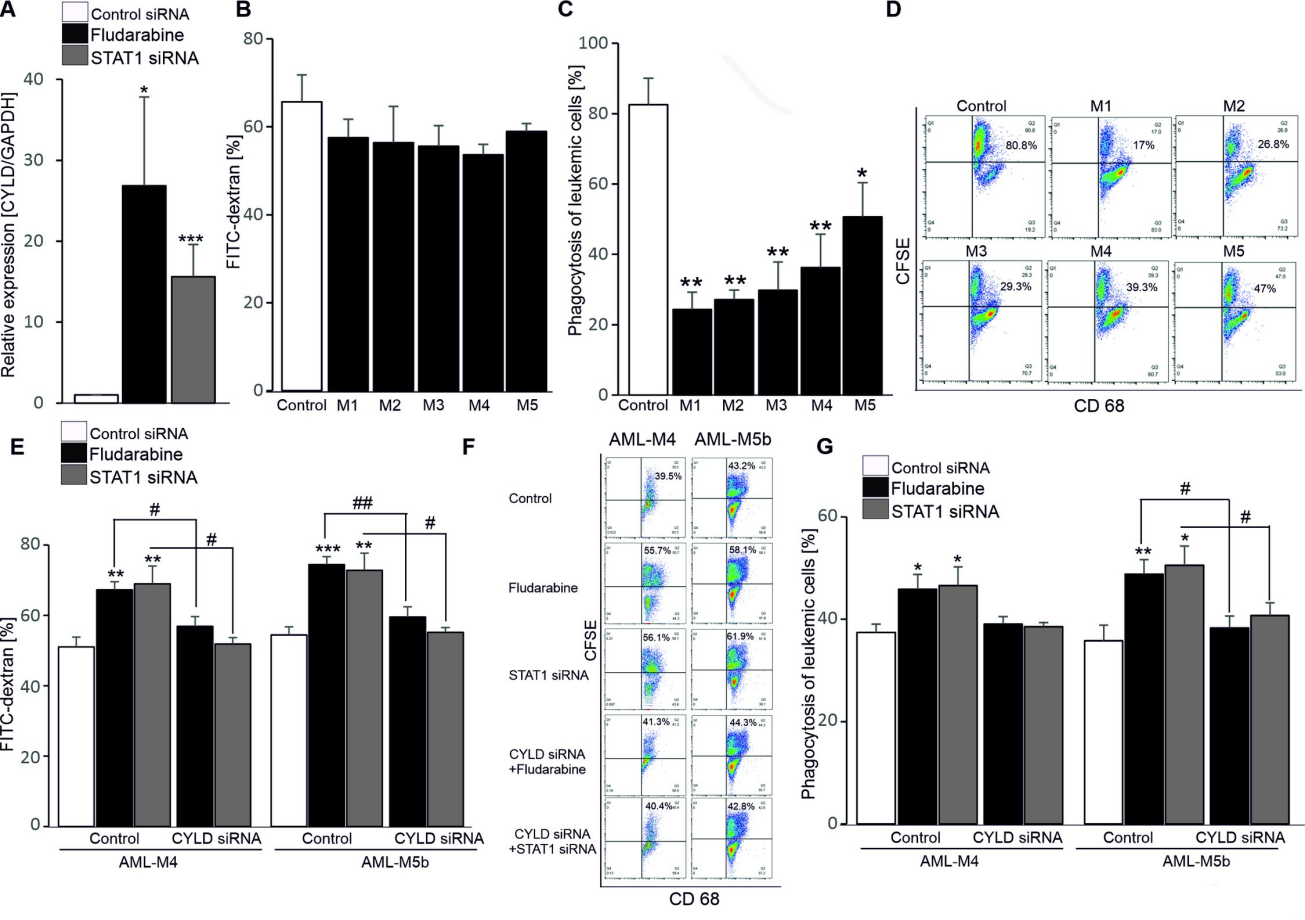
Effects of CYLD on macrophage phagocytosis in AML. A. Arithmetic means ± SEM (n = 6–10) of transcript level of CYLD is shown to control siRNA- (white bar), fludarabine (black bar) and STAT1 siRNA (grey bar)-treated BMDMs. * (p<0.05) and *** (p<0.001) indicate significant differences from controls siRNA-treated BMDMs (ANOVA). B-C. Arithmetic means ± SEM (n = 5 each) of percentages of MDMs from healthy controls (white bars) and BMDMs from AML M1/M2/M3/M4/M5 subtypes (black bars), which were stained by FITC-dextran (B) or positive to CD68 and CFSE (C). *(p<0.05) and ** (p<0.01) indicate significant differences from control MDMs (ANOVA). D. Representative dot plots (n = 5) of CD68+ CFSE+ cells (the phagocytosis of leukemic cells) in MDMs from healthy controls or BMDMs from AML M1/M2/M3/M4/M5 subtypes. E, G. Arithmetic means ± SEM (n = 6–10) of percentages of BMDMs from AML M4/M5b subtype stained by FITC-dextran (E) or positive to CD68 and CFSE (G), which are untreated (white bars) or treated with fludarabine (black bars) or STAT1 siRNA (grey bars) in the absence or presence of CYLD siRNA. *(p<0.05), **(p<0.01) and *** (p<0.001) indicate significant differences from control siRNA-treated BMDMs; #(p<0.05) and ## (p<0.01) indicate significant differences from fludarabine or STAT1 siRNA-treated BMDMs (ANOVA). F. Representative dot plots (n = 6) of CD68^**+**^ CFSE^**+**^ BMDMs from AML M4/M5b subtype, which are untreated or treated with fludarabine or STAT1 siRNA in the absence or presence of CYLD siRNA.

As indicated above, the low CYLD expression was found in 72.83% of the patient group, therefore, involvement of CYLD and STAT1 signalling was examined in macrophages from AML patients. We observed that macrophage counts obtained from AML patients (BMDMs) were comparable to that from healthy donors (MDMs). The CD68 positive rate of BMDMs from all AML subgroups and MDMs were ≥80%. Subsequently, we examined whether treatment of BMDMs with fludarabine or STAT1 siRNA affects CYLD expression. Our data showed that fludarabine or STAT1 silencing significantly enhanced the mRNA level of CYLD in BMDMs (Fig 4A). However, the AML subgroups did not significantly differ in macrophage activation upon knockdown of STAT1, as the amounts of CD11b^**+**^CD68^**+**^ CD86^**+**^ and CD11b^**+**^CD68^**+**^ CD40^**+**^ cells in AML subgroups remained unchanged in the presence of fludarabine or STAT1 siRNA. Similarly, the release of the cytokines TNF-α, IL-6, IL-1β and IFN-γ by BMDMs from the AML subtypes were unaltered when exposed to fludarabine or STAT1 siRNA. The evidences supported that there was no connection of CYLD levels and inflammatory responses in AML patients.

Furthermore, we asked whether FITC-dextran uptake and phagocytosis of leukemic cells are different among the groups. As shown in Fig 4B, FITC-dextran uptake by BMDMs from the five AML subgroups was comparable to that of normal macrophages. Importantly, phagocytosis of leukemic cells by BMDMs from the five AML subgroups was significantly lower than that by MDMs (Fig 4C and 4D), demonstrating that macrophages from AML cells were defect in phagocytosis of leukemic cells.

To test whether knockdown of STAT1 improves phagocytic activity of BMDMs from the different subgroups of AML, the cells were treated with fludarabine or STAT1 siRNA in the presence or absence of CYLD siRNA. Interestingly, FITC-dextran uptake by fludarabine-or STAT1 siRNA-treated BMDMs from M4/M5b, but not M1/M2/M3/M5a subgroup was significantly increased as compared to control MDMs, the effects were reversed in the presence of CYLD siRNA (Fig 4E). Differently, treatment with the STAT1 inhibitor fludarabine or STAT1 siRNA significantly stimulated the elimination of leukemic cells through the expression of CYLD by BMDMs from M5b and partly increased by that from M4 subgroup (Fig 4F–4G). However, knockdown of STAT1 had little effect on the phagocytic activity of leukemic cells by BMDMs from AML-M2/M3/M5a or MDMs.

In addition, survival rate was similar to BMDMs from the five AML subgroups compared to control MDMs in the presence of fludarabine or STAT1 siRNA. Collectively, these data revealed that CYLD sensitive BMDMs from M4/M5b subtype had eliminating effects of leukemic cells in the response to knockdown of STAT1.

## Discussion

CYLD is considered as a negative regulator of hyperresponsive inflammation in macrophages through NF-κB [26] and STAT1 [15] pathways in mice. Similarly, we observed that CYLD inhibited maturation and cytokine secretion as well as induced phagocytosis and apoptosis of human macrophages though STAT-1 signalling. In the absence of CYLD, the expression of costimulatory molecules CD40 and CD86 and the release of IL-6 by MDMs were significantly enhanced, whereas CYLD-silenced MDMs exhibited FITC dextran uptake and the number of Annexin V^**+**^PI^**-**^ cells less than control MDMs. The inhibitory effects of CYLD on human macrophage functions were dependent on activation of STAT1 signalling, which was revealed for the first time in this study.

Moreover, down-expression of CYLD is known to correlate with several blood cancers [27,28]. In consistent, CYLD expression was significantly lower in subtypes with monocytic/myelomonocytic component M4/M5, but not M1/M2/M3 in comparison with healthy controls. Importantly, the frequency of M5a group with the low CYLD expression was 100% and its level was even significantly lower in the M5a compared to M5b groups. Other investigations reveal that inactivated expression of CYLD strongly promotes the Wnt signaling and is associated with a poor prognosis in multiple myelomas [13] and leukemia/lymphoma [11,14]. In addition, the high expression of CYLD have better overall survival in chronic lymphocytic leukemia [14]. Recently, mutations in *CYLD* gene are associated with the susceptibility to pediatric lymphoblastic B-cell leukemia [29], although this gene alterations involved in AML is not fully understood. The results suggested that AML patients with the low CYLD expression had a significant higher relative risk of poor clinical outcomes than those with the high CYLD expression.

Importantly, the low CYLD expression was significantly higher in elderly than young patients and related to the release of LDH in patients with AML, pointing out an involvement of CYLD in metabolic activities in AML patients. LDH is known to catalyse the conversion of pyruvate to lactate, which is associated with tumour metabolism [8] and exerts an immunosuppressive effect in hematologic neoplasms [9]. Lactate inhibits cytotoxic activity of natural killer cells [30] and induces macrophage activation [31]. In this finding, the low CYLD expression enhanced functional activation of human macrophages, suggesting a significant correlation among the expression levels of LDH and CYLD and macrophage activation in AML patients.

Next, the regulatory role of CYLD on functions of macrophages from AML patients was analysed. As known above, CYLD played an important role in modulating activation and maturation of human macrophages through STAT-1 signaling. Moreover, the mRNA level of CYLD was increased upon treatment of BMDMs with STAT-1 signaling inhibitor fludarabine or STAT1 siRNA, we asked therefore whether STAT1 signalling is related to the inhibitory role of CYLD on functions of BMDMs from AML patients. Unlike MDMs, the enhanced expression of CYLD by fludarabine or STAT1 silencing only affected FITC-dextran uptake and the phagocytosis of leukemic cells and unaltered maturation and cytokine productions of BMDMs from AML patients, although knockdown of STAT1 by fludarabine or STAT1 siRNA induced the apoptosis of all MDMs and BMDMs. As indicated in this study, there were marked differences between functional activation of BMDMs from M4/M5b and the other subtypes. Fludarabine or STAT1 silencing stimulated FITC-dextran uptake by BMDMs from M4/M5b subgroup through the expression of CYLD. Moreover, treatment with the STAT1 inhibitor fludarabine or STAT1 siRNA significantly enhanced the elimination of leukemic cells through the expression of CYLD by BMDMs from M5b and partly from M4 subgroup (Fig 4E–4G). Differently, macrophage phagocytosis of acute monocytic leukemia (M4) is known to be dependent on CD47 activation [20] and the chemotactic responsiveness effects of M4 and M5b, but not M5a leukemic cells are comparable to that of normal monocytes [32]. The involvement in macrophage phagocytosis differentiated from M4/M5b subgroup was also reported for the first time to be sensitive to CYLD through STAT1 signalling. Unlike the effects of STAT1 on regulating macrophage functions in AML, STAT3 and STAT5 are illustrated to positively regulate proliferation and anti-apoptotic signals in haematological malignancies [33,34].

In addition, to ask whether CYLD and STAT1 physically interact in MDMs and BMDMs, we immunoprecipitated CYLD from untreated and LPS-treated macrophages. Similar to a recent study in mouse macrophages [15], we found that CYLD did not interact with STAT1 as assessed by Western blot analysis. Therefore, activation of STAT1 may lead to indirect inhibition of CYLD expression.

This finding is supported by other reports indicating that CYLD exerted pro-phagocytic role of leukemic cells by macrophages from M4/M5b subgroup via STAT-1 pathway. Therefore, our findings may provide a new insight for better understanding the promoting effects of CYLD on macrophage phagocytosis in AML upon treatment with fludarabine.

## Supporting information

S1 Fig Effects of CYLD on STAT3, STAT5, STAT6, SHP1 and SHP2 signallings in MDMs. Graphs indicate the mRNA levels of STAT3, STAT5, STAT6, SHP1 and SHP2 in control siRNA- and CYLD siRNA-treated MDMs (n = 8–10), which were unstimulated or stimulated with LPS.
(TIF)

S2 Fig Effects of CYLD on the release of TNF-α, IL-1β and IFN-γ by MDMs. Graphs indicate concentrations of TNF-α, IL-1β and IFN-γ secreted by control siRNA- and CYLD siRNA-treated MDMs (n = 8–10), which were unstimulated or stimulated with LPS.
(TIF)

S3 Fig Effects of CYLD on the phagocytosis of leukemic cells by MDMs. Graph indicates the phagocytosis of leukemic cells by control siRNA- and CYLD siRNA-treated MDMs (n = 7), which were unstimulated or stimulated with LPS.
(TIF)

S1 Dataset (XLSX)
