# Supplementary figures and images for: CYLD stimulates macrophage phagocytosis of leukemic cells through STAT1 signalling in acute myeloid leukemia
Source: PLoS One. 2023 Aug 7;18(8):e0283586. doi: 10.1371/journal.pone.0283586 (PMC10406188; doi:10.1371/journal.pone.0283586)

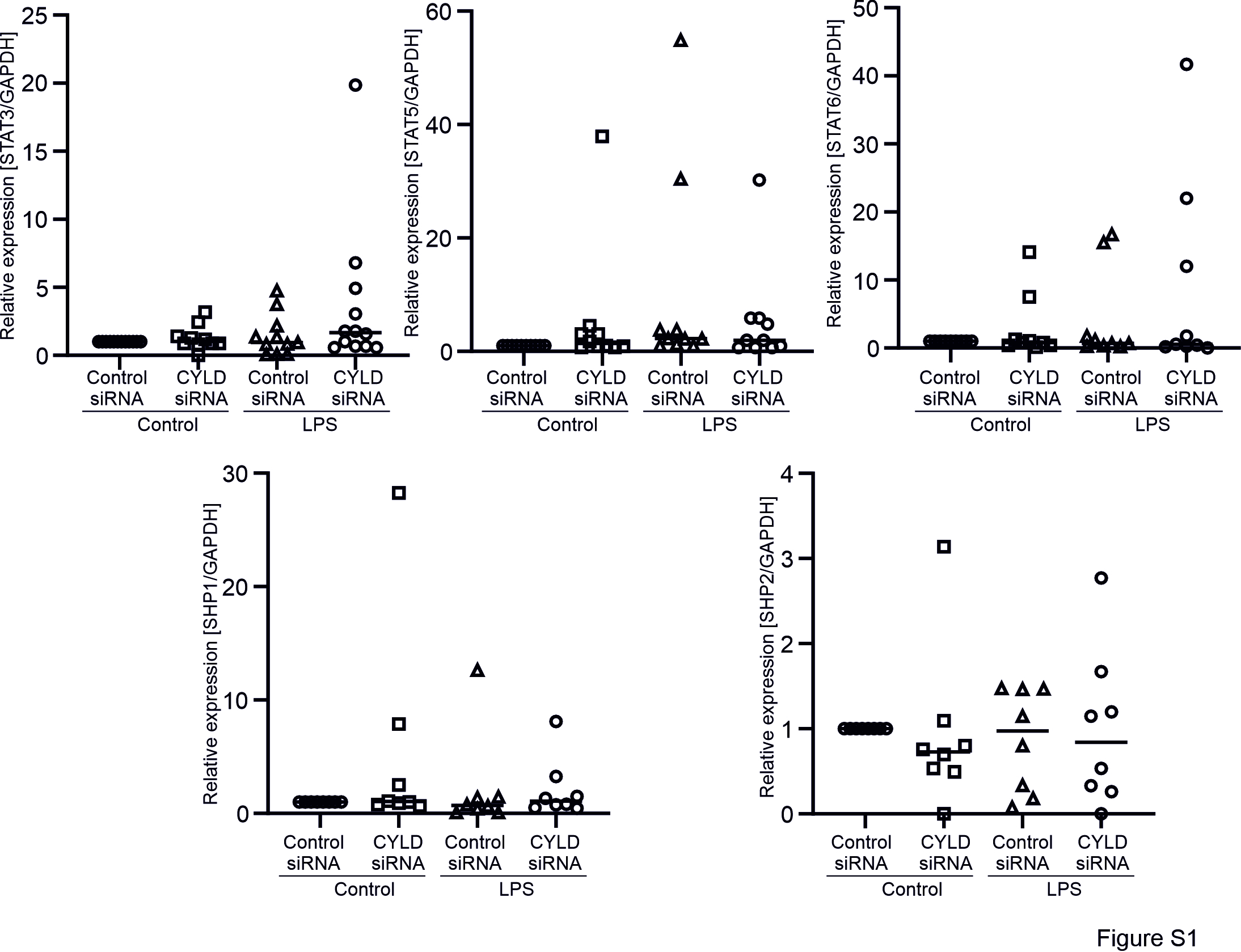

Supplement: S1 Fig — Graphs indicate the mRNA levels of STAT3, STAT5, STAT6, SHP1 and SHP2 in control siRNA- and CYLD siRNA-treated MDMs (n = 8–10), which were unstimulated or stimulated with LPS. (TIF) [file pone.0283586.s001.tif]

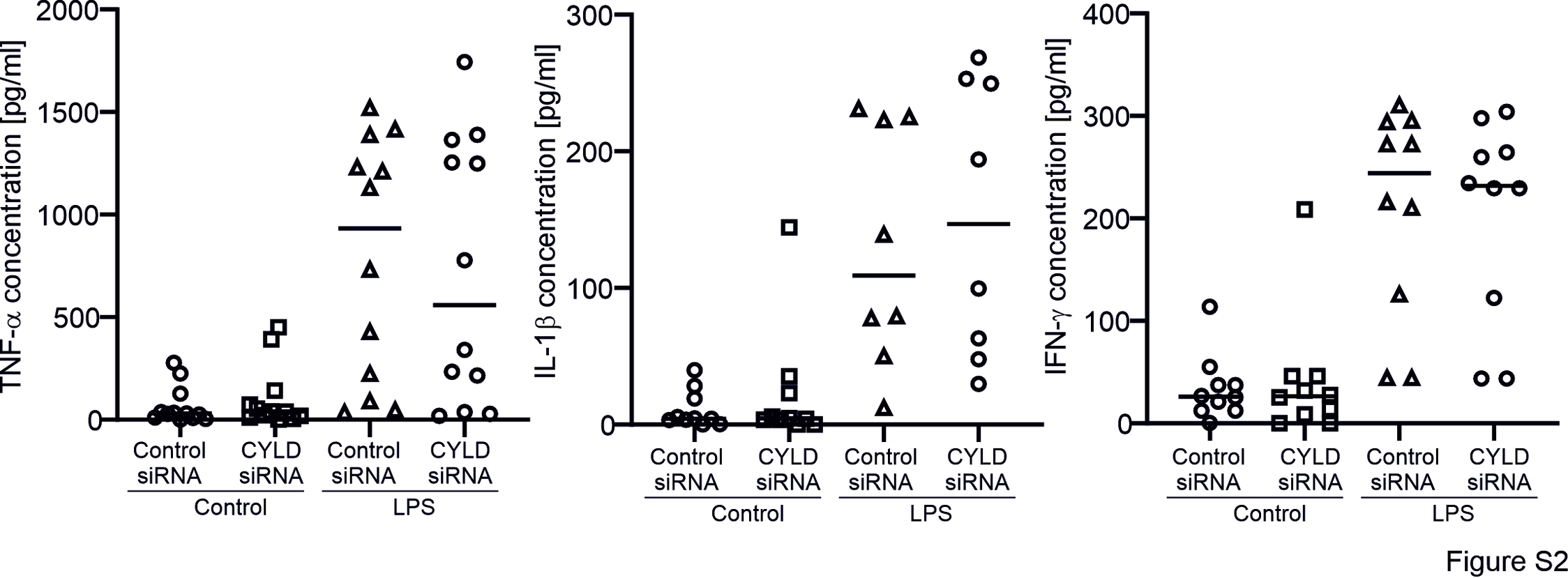

Supplement: S2 Fig — Graphs indicate concentrations of TNF-α, IL-1β and IFN-γ secreted by control siRNA- and CYLD siRNA-treated MDMs (n = 8–10), which were unstimulated or stimulated with LPS. (TIF) [file pone.0283586.s002.tif]

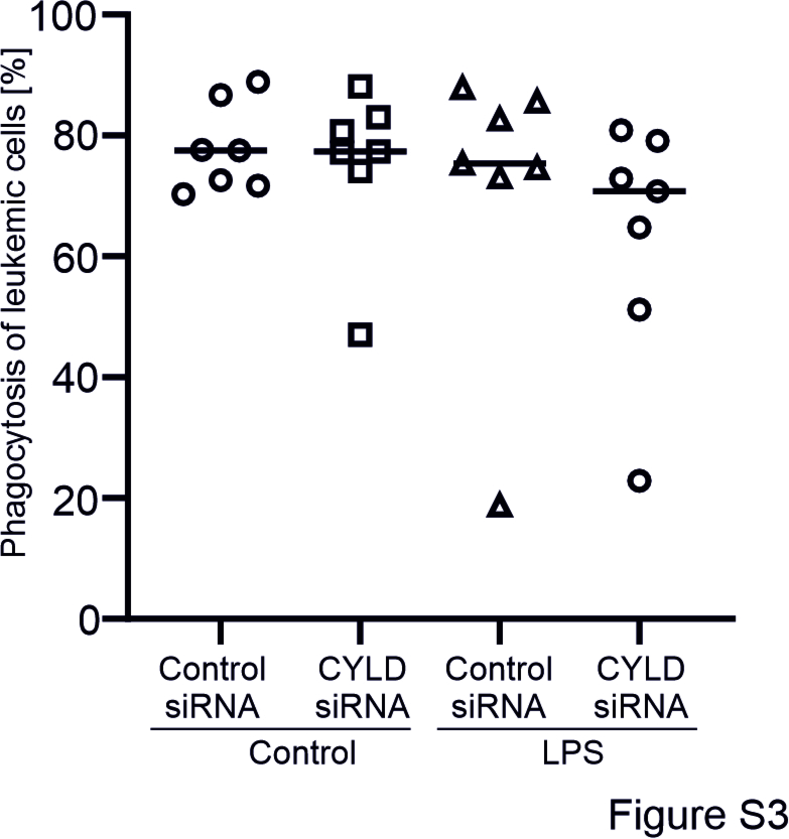

Supplement: S3 Fig — Graph indicates the phagocytosis of leukemic cells by control siRNA- and CYLD siRNA-treated MDMs (n = 7), which were unstimulated or stimulated with LPS. (TIF) [file pone.0283586.s003.tif]
